# Understanding the interplay between extracellular matrix topology and tumor-immune interactions: Challenges and opportunities

**DOI:** 10.18632/oncotarget.28666

**Published:** 2024-11-07

**Authors:** Yijia Fan, Alvis Chiu, Feng Zhao, Jason T. George

**Affiliations:** ^1^Department of Biomedical Engineering, Texas A&M University, College Station, TX 77843, USA; ^2^Translational Medical Sciences, Texas A&M University Health Science Center, Houston, TX 77030, USA; ^3^Center for Theoretical Biological Physics, Rice University, Houston, TX 77005, USA; ^4^Department of Hematopoietic Biology and Malignancy, MD Anderson Cancer Center, Houston, TX 77030, USA

**Keywords:** ECM, tumor-T cell evolution, tumor microenvironment

## Abstract

Modern cancer management comprises a variety of treatment strategies. Immunotherapy, while successful at treating many cancer subtypes, is often hindered by tumor immune evasion and T cell exhaustion as a result of an immunosuppressive tumor microenvironment (TME). In solid malignancies, the extracellular matrix (ECM) embedded within the TME plays a central role in T cell recognition and cancer growth by providing structural support and regulating cell behavior. Relative to healthy tissues, tumor associated ECM signatures include increased fiber density and alignment. These and other differentiating features contributed to variation in clinically observed tumor-specific ECM configurations, collectively referred to as Tumor-Associated Collagen Signatures (TACS) 1–3. TACS is associated with disease progression and immune evasion. This review explores our current understanding of how ECM geometry influences the behaviors of both immune cells and tumor cells, which in turn impacts treatment efficacy and cancer evolutionary progression. We discuss the effects of ECM remodeling on cancer cells and T cell behavior and review recent *in silico* models of cancer-immune interactions.

## INTRODUCTION

The immunosurveillance hypothesis proposes that the immune system serves as a barrier against cancer progression by targeting tumor-associated antigens (TAAs) [1, 2]. This process, called cancer immunoediting, acts as an evolutionary filter that either eliminates cancer cells or permits their escape and leads to clinically significant disease. Cancer immunoediting involves three phases: elimination, equilibrium, and escape [2, 3]. While empirical evidence on early tumor-immune interactions is limited, theoretical models suggest that healthy adaptive immune systems can control many cancer initiation events [4, 5]. Stochastic dynamical modeling has previously modeled cancer incidence resulting from fully immune-evasive cancer subclones and predicts diverse TAA distributions following tumor immune escape [6, 7]. In addition to the generation and presentation processes of TAAs, another potential cause of tumor evasion is insufficient T cell interaction, often a result of immune cell exclusion in solid tumors [8–11]. In some cases, there is a complete lack of T cells in the tumor vicinity, referred to as an immune desert [9]. These scenarios contrast with “immune-inflamed” disease, wherein significant immune cell infiltration into the tumor core and subsequent activation is observed [9, 11]. Hence, tumors can be categorized into “cold” or “hot” based on the extent of infiltration and cytotoxicity of cytotoxic immune cells within the TME. The underlying mechanisms and the transformation from cold to hot, have received significant research attention [9, 12, 13].

**Figure 1:**
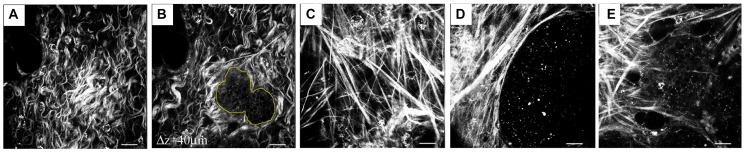
Representation of common TACS in mouse mammary tissue. (**A**, **B**), TACS1, (**C**, **D**): TACS2, (**E**). TACS3 (images obtained from [47]).

Numerous factors can result in insufficient immune infiltration, such as abnormal vasculature, hypoxia, defective T cell priming, and ECM topology [9, 10, 14, 15]. As one of the most abundant components in the TME, the ECM plays a critical role in regulating cell growth, survival, and differentiation [16]. In the context of cancer, the ECM undergoes significant changes relative to that of normal tissue, such as increased synthesis, accumulation, and alignment [17]. The most commonly observed geometric configurations, known as Tumor-Associated Collagen Signatures (TACS), were first identified in 2006 in the Wnt-1 mouse mammary tumor model [17]. In this model, dense type-I collagen fibers were found in tumor-bearing mice, leading to the characterization of three distinct TACS types: TACS-1, TACS-2, and TACS-3 (Figure 1) [17]. Subsequently, TACS1-TACS3 have been observed in human breast cancer and pancreatic adenocarcinoma [18–20]. In many other cancer types (as detailed in Table 1), dense accumulations of not only collagen but also fibronectin and laminin have been reported [21–26]. Significant research has been directed at exploring the interplay between tumor-specific ECM fiber characteristics - including stiffness, density, and topological changes - and their impact on tumor progression, metastasis, and immune function [18, 27–31]. This review focuses on the impact of variable TACS on the behaviors of both tumors and T cells and the interactions between them. Understanding the complex interplay is relevant for developing more accurate model of tumor evasion and the identification of corresponding therapeutic intervention.

**Table 1:** The impact of TACS on tumor cells and T cells in various tumor types

Tumor type	Main findings
Lung cancer	T cells exhibited limited migration in regions characterized by high matrix density. The aligned fibers present in perivascular regions and surrounding tumor epithelial cell areas determined the migratory path of T cells, constraining their entry into tumor islets [15]. Resident CD8+ T cells tend to accumulate in the stroma and move slowly within this area [84].
Ovarian cancer	Space constraints restrict the movement of CD8+ T cells within the tumor stroma, causing them to migrate with alternating forward and backward movements [84]. A fibrous stroma can function both as a physical barrier and as a guiding framework for resident CD8+ T cells [84].
Breast cancer	The presence of TACS3 is associated with poor outcomes in both disease-specific and disease-free survival [18, 19]. TACS is expected to provide insights into the invasive potential of tumors, making it a valuable tool for identifying and characterizing breast tumors in both animal and human tissues [17]. Collagen alignment quantification is a promising and innovative approach for predicting the survival outcomes in human breast cancer [18, 49]. Collagen alignment and stromal syndecan-1 expression did not serve as predictors for ductal carcinoma *in situ* (DCIS) recurrence. However, there was a higher frequency of collagen fibers perpendicular to the duct perimeter in DCIS lesions with characteristics typically associated with a poor prognosis [29].
Melanoma	Age-associated ECM alterations may promote the migration of melanoma while simultaneously impeding the infiltration of immune cells [120].
Pancreatic adenocarcinoma	The collagen surrounding malignant ducts displays elevated levels of alignment, increased length, and greater width compared to both normal ducts and benign ducts [121]. Dense collagen networks serve as a physical barrier, further reorganizing the distribution of T cells to favor the tumor stroma. These mechanisms primarily contribute to the entrapment of intratumoral T cells in pancreatic cancer [86]. TACS plays a role in the early dissemination of histologically premalignant lesions and ongoing invasion from well-differentiated disease. TACS has the potential to be used as a biomarker for enhancing the pathological evaluation of early-stage disease [20].
Renal cell carcinoma	There was a significant increase in collagen density and alignment observed in grade 4 renal cell carcinoma (RCC) compared to grade 1 RCC [122].
Gastric carcinoma	Elevated levels of collagen in the tumor stroma act as a hindrance to the infiltration of CD8+ T cells and may represent a mechanism by which tumors evade host immune responses [85].

## ABNORMAL ECM IN TUMORS

The ECM comprises an elaborate collection of biomacromolecules, including proteins, glycosaminoglycans, proteoglycans, and polysaccharides, which form a network of polymeric nanofibers [16, 32]. This network gives rise to the ECM’s viscoelastic properties, enabling the ECM to undergo deformation and remodeling for critical functions in tissue development, wound healing, and maintenance [32–34]. In many disease states, including cancer, the ECM undergoes pathological remodeling. Although the early stages of cancer growth sometimes resemble wound healing, tumors can modify the surrounding ECM in ways that are distinct from the wound healing processes. For this reason, tumors are often referred to as “unhealed wounds” [35]. Specifically, unlike the repair process in healthy tissue, which generates curved and relaxed ECM fibers, tumor remodeling results in matrix fibers that are thicker, stiffer, and more aligned [17, 31].

These morphological differences can be attributed to a combination of mechanical and biological cues. Specifically, cancer cells may undergo an Epithelial-to-Mesenchymal Transition (EMT), which plays a significant role in disrupting tension homeostasis and is a characteristic feature of various tumors [36–39]. Moreover, phenotypically plastic stromal cells, including Cancer-Associated fibroblasts (CAFs), they can also be reprogrammed by tumor cells, such as highly migratory tumor cells and cells that have undergone partial EMT, potentially leading to changes in tumor cell behavior [40, 41]. Through these interactions, the cancer cells generate traction forces, exerting mechanical stress on the ECM [42, 43]. These traction forces exerted by cancer cells can remodel the geometry of the ECM in two ways. Firstly, they compact the ECM fibers towards the cell, resulting in denser, stiffer, and straighter fibers [17, 31]. Secondly, these forces can indirectly influence the surrounding stromal cell alignment, which in turn remodel and redeposit the ECM [17]. In addition to mechanical forces, *in vitro* experimental evidence also indicates that the secretome of cancer cells can independently contribute to ECM fiber alignment [44]. CAFs also influence fiber reorganization by expressing various cytokines including Discoidin Domain Receptor (DDR)1, DDR2, Cav-1, and TGF-β [44–46]. Notably, TGF-β plays a significant role by releasing a Cellular Communication Network (CCN), within which WISP1 is highly up-regulated by TGF-β and binds to ECM fibers, inducing their linearization [44]. Consequently, the alignment of ECM within the TME arises from the synergistic effects of multiple distinct processes.

In some tumors, three distinct TACS occur in different disease stages and geographic regions [18, 20]. Upon the appearance of the tumor, there is a significant increase in the density of ECM fibers around the tumor, a phenomenon known as TACS1, which has been observed in both mouse mammary tumors and human breast cancer [17, 18, 47]. This serves as a reliable hallmark for detecting early-stage tumors. Subsequent tumor growth and ECM remodeling lead to the second stage, TACS2, in which collagen fibers become stretched, taut, and parallel to the tumor boundary [17, 47]. TACS2 is primarily believed to occur due to tumor growth, which stretches the stroma, thereby stimulating fibroblasts and constraining portions of the tumor [17]. When tumors are prepared to invade, especially at the tumor boundary and particularly in a collective manner, the ECM undergoes further remodeling, resulting in the formation of TACS3, also known as a radial-packed ECM. In TACS3 invasive regions, the angle of the ECM fibers relative to the tumor boundary typically aligns around 90° [17, 47]. In studies such as automated sequential microprinting of tumor and endothelial cells in ECM scaffolds, quantitative reflection microscopy has shown that both mouse and human breast tumor spheroids induce radial orientation of the surrounding collagen fiber network up to a distance of five times their radius [48]. Therefore, these radial fibers, remodeled by pioneer invasive tumor cells, provide favorable physical conditions for tumor metastasis. Subsequent tumor cells can benefit from this pre-established structure as they invade further, while also remodeling the surrounding fibers. Three TACS architectures in mouse mammary tissue are shown in Figure 1. Subsequently, in studying the invasion front of human breast primary tumors, five new TACS categories (TACS4-8) were refined and introduced based on TACS1-3, considering the clarity of tumor boundaries and the heterogeneity of tumor migration directions [49]. Given that TACS1-3 are the most representative and distinct, our subsequent discussion will focus exclusively on these categories.

It should be noted that: (1) The presence of different TACS is not limited to a specific cancer stage. In breast cancer, the appearance of TACS3 typically occurs at advanced stages and is indicative of tumor cell intravasation, with TACS3-positive patients having a lower survival rate compared to TACS3-negative patients [18, 50]. However, in Pancreatic Ductal Adenocarcinoma (PDA), TACS3 is observed in the early stage of the disease, evidenced by irregular boundaries of ductal structures, and becomes prevalent in the later stages [20]. (2) Different TACS may not necessarily occur in all tumor progression processes. For example, in breast cancer, TACS3 cannot be observed in the pathological slides of all patients [18]. (3) Different TACS can simultaneously appear in various regions. In breast carcinoma, ECM fibers near the tumor-stroma boundary are reorganized into a circumferential pattern (TACS2), while those in the invasive boundary are arranged radially (TACS3) [18]. As the tumor progresses, fibers that were initially packed circumferentially can be remodeled into a radial configuration [18]. In PDA, some regions of the duct are positive for TACS2, while neighboring regions of the duct are positive for TACS3 [20]. Therefore, the evolution of TACS varies depending on the individual and the cancer subtype.

A growing body of evidence indicates that ECM geometry remodeling plays a significant role in regulating both tumor and T cell behaviors [31, 51, 52]. We next discuss the independent effects of TACS on tumor cells and T cells.

## EFFECTS OF ECM REMODELING ON CANCER CELLS

### Tumor heterogeneity

Inhomogeneities in the microenvironment create selective pressures that promote the emergence of differential tumor cell phenotypes and intratumoral heterogeneity [53]. The various aspects of the ECM, such as architecture, composition, and mechanical properties, all influence tumor heterogeneity. These effects are numerous and manifest as feature diversity amongst individual cancer cells in a primary tumor. Relevant features include regulation of nutrient availability, gene expression, and migratory behavior, which all enhance cancer phenotypic heterogeneity [54]. In this context, we focus our discussion on the impact of ECM architecture on tumor heterogeneity.

Based on our current understanding, ECM geometry exerts its influence on tumor heterogeneity primarily in a spatiotemporal manner. Since changes in TACS are spatiotemporally defined [18, 20], interactions between tumors located in different regions and TACS with different properties may affect both the spatial heterogeneity of tumors and ECM properties. For instance, in pathological slices from clinical breast cancer patients and *in vitro* breast cancer cell lines, the invasive front of tumors often exhibits increased ECM fiber alignment, and high stiffness compared to noninvasive tumors [30, 55]. In contrast, central regions of tumors typically exhibit reduced ECM content, less pronounced fiber alignment, and stiffness [17, 30, 55]. It has been shown that in MDA-MB-231 *in vitro* cell culture, ECM fiber alignment at the invasive front triggers mechano-transduction and tumor invasion in a strain-dependent manner [56]. This process in turn increases ECM deposition and stiffness and activates latent growth factors and cytokines stored in the ECM, such as TGF-β1, which can induce fibroblast differentiation [30, 57]. Cryptic binding sites are exposed to soluble factors, such as *F*_N_ *III*_12−14_, and this particular factor has been shown to bind fibroblast growth factor (FGF)-2 and vascular endothelial growth factor (VEGF)-A with high affinity [58–60].

### Tumor invasion and metastasis

Studies using breast carcinoma models, such as Wnt-1 and PyMT, have first shown that the ECM can regulate cell invasion and metastasis [17]. Upon the discovery of different TACS, research has focused on the migration characteristics of tumor cells and T cells within differently aligned ECM. In *in vitro* and *in silico* experiments, researchers have summarized the migration characteristics of cells in randomly packed, horizontally packed, TACS2, and TACS3 ECM architectures [17, 20, 47, 51, 52]. In TACS2, the disseminated human PDA cells adhere to the main duct and exhibit alignment in accordance with TACS2 and are likely to undergo directed motility along the collagen matrix [20]. Moreover, *in silico* simulation shows that high-density aligned fibers could prevent tumor cells from migrating outward [51]. In TACS3, tumor cells exhibit a directed or “fingering” invasion pattern, eventually dispersing into numerous masses through a collective invasion mechanism, a phenomenon further confirmed in human PDA [20, 51]. These peripheral pioneering tumor masses, distinct from the tumor core, usually have undergone EMT and can also spread through the stroma in a directed manner [61]. One *in silico* study has attempted to quantify the invasion advantage of tumor cells in TACS3. They concluded that within TACS3, tumor cells can migrate farther in the same timeframe compared to randomly packed fibers [51]. Collectively, these investigations have demonstrated that the formation of aligned fibers, primarily TACS3, promotes invasion compared to randomly packed and TACS2 fibers and that tumor is highly likely wherever regions with TACS3 are present [17]. Furthermore, from both mouse and human breast tumor samples, remodeling of the ECM fiber architecture is often accompanied by an increase in stiffness [30, 62]. It has been observed that human breast cancer cells migrate more rapidly in stiff ECMs, which is consistent with the fact that aligned fibers contribute to enhanced cell migration efficiency [30, 51]. Additionally, it has been found that *in vitro* human breast epithelial cells primed in a stiff ECM retain this rapid migration mechanical memory and carry it into future ECM environments, continuously maintaining high-speed migration and exhibiting a strong capacity for fiber remodeling [63, 64]. Due to the heterogeneity in the distribution of stiffness within tumors [55], it is possible that tumors in different locations possess varying migration abilities and ECM remodeling capacities. Consequently, it is possible that tumor cells and the surrounding ECM have a mutual influence on each other, collectively forming a spatial heterogeneity configuration.

To investigate the reasons behind the emergence of the “contact guidance” theory or the different migration patterns of cells within various ECM architectures, a study was conducted by culturing human breast and pancreatic carcinoma cells on substrates that simulate the aligned fiber architecture [65]. It was found that the mechanosensitive sensors of tumor cells, specifically Focal Adhesions (FA) [42], become confined and mature in an anisotropic manner. This event in turn, triggers the reorganization of the actin cytoskeleton and promotes directional cell migration [65].

The above studies have established a qualitative link between tumor cell invasion and ECM geometries. There now exists a need for more quantitative studies focused on tumor invasion within different TACS contexts. Such investigations will guide a dynamic understanding of the extent to which TACS influences tumor invasion. TACS states and corresponding transitions are likely more complicated than the prototypical patterns and exist on a spectrum. We thus anticipate that a model taking into account the effects of precise TACS dynamics on tumor behavior and tumor-T cell interactions will be crucial for better understanding the link between TACS and survival.

### Tumor immune detection

T cell-mediated tumor immunogenic identification occurs through the recognition of tumor antigenic peptides presented on the surface of cancer cells [66]. Additionally, these antigens must be made accessible for uptake by Antigen Presentation Cells (APCs) [66]. There is little direct evidence explaining how distinct ECM geometries influence the spatiotemporal tumor immune interactions. A recent theoretical study that considered the diffusion and decay of antigens released by necrotic tumor cells argued that cancer cells in TACS3 tend to release antigens earlier than those within randomly or horizontally aligned ECM structures [51]. This finding suggests that ECM structures need not necessarily block tumor antigen secretion outright – but may instead cause a time shift in the process – in order to influence the tumor-immune interaction. Presumably, cancer populations growing in high-surface-area configurations have greater selective pressure on their antigen expression and secretion because of more interaction with T cells, although these dependencies require further investigation. Additionally, many *in vitro* studies have established a correlation between ECM stiffness and tumor cell proliferation [55, 67, 68]. It is well known that increased ECM stiffness is typically associated with higher levels of fiber alignment [30, 62]. Hence, these observations suggest a potential association between ECM alignment or stiffness and tumor cell fitness. Notably, the impact of ECM stiffness appears to vary depending on the tumor type. For instance, in glioma cancer, increased ECM rigidity has been associated with enhanced cancer cell migration and proliferation [69]. Conversely, in breast cancer, elevated ECM rigidity promotes cancer cell migration while inhibiting proliferation [55]. This observation is supported by findings indicating that matrix rigidity modulates TGFβ1-induced EMT and apoptosis, where EMT facilitates tumor cell migration [67, 68]. In hepatocellular carcinoma (HCC), the increase in matrix stiffness has been found to induce drug resistance by regulating the PI3K and ERK signaling pathways [70].

In summary, TACS and tumors mutually influence one another through various cellular and mechanical mechanisms. Tumor progression leads to spatial variations in TACS development. Conversely, the spatial distribution heterogeneity of TACS also shapes interactions with tumors, influencing tumor adaptability and the spatial complexity of tumor progression. Thus, owing to its spatial and temporal dynamics, TACS plays a regulatory role within the TME, influencing not only the physical arrangement of tumor cells but also other TME constituents. These interactions therefore have the potential to modulate dynamics within the TME and require further research to study which features can serve as potential therapeutic targets.

## EFFECTS OF ECM REMODELING ON T CELL BEHAVIOR

### T cell activation

T lymphocyte precursors initially originate within the bone marrow and then migrate to the thymus, where they further develop into either CD4+ or CD8+ lineages [66]. Following a rigorous selection process, T cells are regarded as naïve until they encounter their specific antigen via the T cell receptor (TCR) and receive co-stimulation from APCs [66]. This activation triggers a transition to an effector cell state [66], wherein T cells are ready to recognize and engage with foreign peptide signatures through TCR binding. While there is limited research on the relationship between ECM geometry and T cell activation, it is known that TCRs are highly sensitive to mechanical forces and can adapt their behavior accordingly [71]. Furthermore, it is known that increased ECM stiffness can lead to impaired TCR activation, and increased stiffness often coexists with alignment [30, 71–73]. Therefore, it is possible that due to the spatial heterogeneity of ECM alignment and stiffness, T cell activation also exhibits certain regional heterogeneity.

### T cell infiltration

Naïve T cells migrate between lymphoid organs via a Brownian-like or subdiffusive random walk and respond to the chemokine CCL21 to regulate their entry into or exit from the bloodstream and lymph nodes, thereby enhancing their chances of encountering APCs [74–76]. In contrast, activated T cells adopt directional migration guided by chemotactic cues within the lymph node [76]. In peripheral tissues, T cells exhibit a super-diffusive, Lévy-type walk, using higher speeds and turning behavior to optimize scanning area [76]. In healthy and tumor tissues, T cell movement depends on phenotype, which includes the amoeboid phenotype with low adhesion pseudopodia and the more mesenchymal-like phenotype with adhesive spreading. This phenomenon, known as amoeboid-mesenchymal plasticity, allows T cells to switch between phenotypes for efficient navigation through heterogeneous conditions [77]. Meanwhile, T cell movement within the TME also adheres to the “contact guidance” theory [78]. However, unlike tumor cells, T cells are additionally influenced by chemotaxis [79].

Wilkinson and colleagues were the first to note that crosslinked ECM fibers influence the migration of lymphocytes *in vitro* [78]. Following this discovery, there has been considerable research on the role of ECM fibers in directing T cell migration [80–82]. One continuing debate involves the extent to which the ECM serves as an obstacle to T cell infiltration. To date, numerous *in vitro* and *in silico* studies have shed light on the performance of T cells within TACS3, primarily manifested as aligned collagen fibers can enhance T cell motility, resulting in faster, straighter, and further migration paths [31, 51, 83]. Intriguingly, in one *in silico* study, the total number of activated T cells entering the tumor regions is higher in TACS3 when compared to random and aligned ECM fibers [51]. The rapid, direct, and prolonged migration observed can be attributed to morphological changes in T cells within an aligned ECM environment, characterized by elongation and a reduction in the number of protrusions [83]. In combination with their high directional persistence, T cells can cover greater distances when migrating within an aligned ECM [83]. This highlights the crucial role that TACS3 plays in enhancing T cell migration efficiency. It is likely that the debate regarding the hindrance of T cell infiltration by ECM may pertain more specifically to TACS3-negative regions, such as TACS2. For example, studies found that in ovarian, lung, gastric, and pancreatic adenocarcinoma, T cells migrate along TACS2 and are trapped in the stroma, rarely found in the tumor epithelial region [84–86]. One plausible biochemical explanation for this phenomenon could be the role of DDR1, a collagen receptor with tyrosine kinase activity. DDR1 has been implicated in promoting immune exclusion by inducing the alignment of collagen fibers, as demonstrated in Triple-Negative Breast Cancer (TNBC) [45]. Their study revealed that ablating DDR1 in TNBC mouse models enhanced intratumoral T cell infiltration and suppressed tumor growth [45]. Another reason for T cells becoming trapped in the tumor stroma is the activation of CAFs [8, 87, 88]. In the PyMT cancer model, it has also been found that collagen XII, secreted by CAFs, can regulate the assembly and spatial organization of collagen I, thereby facilitating cancer cell invasion and metastasis [62]. In human Non-Small Cell Lung Cancer (NSCLL), two specific CAF populations-FAP^+^αSMA^+^ CAF and MYH11^+^αSMA^+^ CAF-have been found to be associated with regions of T cell exclusion [89]. However, both *in vitro* experiments and *in silico* studies have demonstrated that this physical barrier is not an insurmountable obstacle for the T cells to infiltrate. A study demonstrated that in the presence of CCL5 within tumor islets, a known attractant for T cell migration [90], despite the stroma showing a fivefold higher T cell infiltration per unit volume compared to the tumor islets, T cells were still able to infiltrate into the tumor islets [15]. Another study found no discernible correlation between cluster-level infiltration patterns and typical ECM properties capable of influencing motility [91].

Future work on the impact of TACS on T cell infiltration would benefit from closing two key gaps. Firstly, exploring the extent to which TACS influences T cell infiltration requires careful investigation. Secondly, while we understand that increased and more effective T cell infiltration is typically associated with better prognosis in many cancer [92–94], and more active T cells are found in radially packed ECM architectures [51], lower survival rates are commonly observed in TACS3 positive patients, especially in breast cancer [18]. Additional efforts should aim to elucidate the relationship between decreased survival rates and TACS3, to attempt to decouple the two.

### T cell recognition and killing

As T cells encounter tumors, they need to recognize TCR-specific antigens presented on MHC-I molecules on the tumor surface [66]. Once the antigen is recognized, T cells bind to the target cells and establish an immunological synapse to deliver death ligands and cytotoxic granules, inducing apoptosis of the target cells [66]. Currently, there are several opinions regarding the influence of ECM on T cell cytotoxicity. T cells cultured on matrices with higher collagen concentrations exhibited reduced efficacy in killing autologous melanoma cells [95]. Acknowledging that higher density corresponds to increased stiffness [30], another study that examined T cell behavior on substrates with varying stiffness levels found that T cells exhibited heightened effector functions on stiffer substrates [96]. Table 1 summarizes our current understanding of the impact of TACS on the behaviors of tumors and T cells in various tumor types.

## *IN SILICO* ECM AND TUMOR-T CELL INTERACTION MODELS

A number of computational models have been developed in recent years to link underlying biophysical mechanisms with observed dynamics. Many of these models assume the “contact guidance” theory [78, 97]. The earliest computational model elucidating the interaction between the ECM and tumor cells was developed in 2008 [98]. This study employed the Cellular Potts Model based on the differential adhesion hypothesis, where cells evolve to minimize lattice energy, revealing that glioma cell invasion is enhanced under low collagen concentration and high alignment conditions [98]. Subsequently, several modeling strategies emerged, which we discuss below.

The first modeling approach describes the interactions between cells and the ECM at the cellular level. For example, one study developed a computational framework for characterizing invadopodia protrusions, allowing two-way interactions between the intracellular branched actin network and the ECM fiber network [99]. Other studies employed the finite element method to develop models that elucidated key mechanobiological mechanisms such as actin cytoskeleton contraction during cell-matrix interactions [100–104]. Additional models have simulated the bidirectional interactions between filopodia and the ECM under the influence of both mechanistic and chemical factors. These studies demonstrated that cells can sense the stiffness of the surrounding matrix and confirmed the durotaxis mechanism [43, 105].

The second type of model simulates cell behavior under specific ECM conditions. These ECM characteristics typically include stiffness, alignment, viscosity, and porosity. For example, in one study, a continuum porous media model was developed, concluding that matrix stiffness and viscosity are negatively correlated with tumor growth, while matrix porosity favors tumor growth [106]. Another study utilized the Cellular Potts Model to simulate the behavior and potential interactions between tumor and immune cells under varying ECM densities and morphologies. They found that higher ECM density hinders the migration of both tumor cells and T cells, as well as the conversion of T cells to cytotoxic T cells, thereby affecting potential tumor-T cell interactions. In radially aligned ECM, both tumor and T cells face the least movement restrictions compared to random and horizontally aligned ECM [51]. This topic is not solely focused on tumor cells. More broadly, as the ECM plays a critical role in processes such as embryonic development and wound healing, one study has developed individual-based modeling framework to comprehensively investigate single-cell migration influenced by force-based mechanisms, contact guidance, and matrix remodeling [107]. Specifically, in the context of wound healing, one study utilized a multiscale approach to simulate fibroblast migration within wound tissue. This analysis reported a rapid influx of fibroblasts into the wound space that results from chemoattraction and reorientation/interdigitation of the collagen matrix during healing [108].

The third type of model extends the second by incorporating the dynamic transitions between different TACS. A notable study employed a multi-cellular lattice-free agent-based model to simulate these dynamic transitions [109]. Lastly, and given the important role of the ECM in cancer progression and prognosis, numerous models have emerged in recent years to automatically extract fiber characteristics such as alignment, texture, density, and thickness from pathology slides [110–114].

## CONCLUDING REMARKS AND FUTURE OUTLOOK

Over two decades ago, it was discovered that T cell infiltration can serve as a prognostic indicator across various cancer subtypes, such as breast cancer, ovarian cancer and colorectal cancer, with higher levels of infiltration generally associated with better patient prognosis [115–117]. However, T cell infiltration often exhibits considerable spatial heterogeneity and not all cancer subtypes exhibit therapeutically significant T cell infiltration [118]. As a result, extensive research efforts have focused on the reasons for T cell exclusion and also inadequate recognition. Following the development of the “contact guidance” theory of T cell movement and the discovery of different TACS configurations [17, 119], one possibility was that the TACS signatures, which in the case of TACS2 forms multiple layers of fibrous networks resembling physical walls around tumors, might impede the encounter and interaction between tumors and T cells [84, 86]. This hypothesis led to initial investigations into the impact of ECM architecture or TACS on tumor and T cells, specifically focusing on the impact of TACS on tumor and T cell migration, or whether TACS acts as a physical barrier to T cell infiltration. In these studies, the phenomenon of “contact guidance” of both tumor cells and T cells was confirmed in various cancer types through both *in vitro* and *in silico* studies [20, 31, 47, 51, 52, 83]. Evidence supporting the ECM as a physical barrier to T cell infiltration was obtained in mouse models where collagen alignment, influenced by DDR1, is correlated with reduced T cell infiltration [45]. When DDR1 was removed, increased T cell infiltration and reduced tumor size were observed [45]. The alternative view is that the ECM may impede, but not entirely block, T cell infiltration. This position is supported by observations that infiltrated T cells were still found in aligned regions of the ECM, albeit in smaller numbers [15, 90, 91].

This question of the relative influence of the ECM on T cell infiltration hindrance bears clinical significance as it influences effective tumor treatment strategies and the extent of immunoediting that may occur prior to treatment. Such questions, which are difficult to parse in largescale *in vitro* studies, provide exciting opportunities for mathematical models to efficiently simulate many cases. Model construction and implementation are expected to improve our quantitative understanding of heterogeneous cancer progression, and they can also generate informed predictions from a high-dimensional search space that can guide informed experimental follow-up. More specifically, future research efforts directed at quantifying the effects of TACS on tumor and T cell migration will improve our understanding of whether and to what extent TACS modulates the tumor-T cell interactions. Such future follow-up may include an examination of how TACS influences the spatiotemporal dynamics of tumor-T cell interactions, as well as a characterization of the potential phenotypic changes, evolution of tumor and T cells, and the potential implications for patient survival. Future efforts should also incorporate the relationship between the ECM and additional cellular features, including the role of various cell types that rely on ECM for migration, cells that may alter ECM alignment or remodeling, and the influence of the ECM on various tumor metabolic processes. Given the potential significance of TACS within the TME, future directions should also investigate how ECM-modifying therapies can be more effectively utilized to dynamically control cancer progression with the aim of improving patient survival.
